# Oral or transdermal opioids for osteoarthritis of the knee or hip

**DOI:** 10.1590/S1516-31802009000600015

**Published:** 2010-05-21

**Authors:** Eveline Nüesch, Ane W. S. Rutjes, Elaine Husni, Vivan Welch, Peter Jüni

## Abstract

**BACKGROUND:**

Osteoarthritis is the most common form of joint disease and the leading cause of pain and physical disability in the elderly. Opioids may be a viable treatment option if patients suffer from severe pain or if other analgesics are contraindicated. However, the evidence about their effectiveness and safety is contradictory.

**OBJECTIVES:**

To determine the effects on pain and function and the safety of oral or transdermal opioids as compared with placebo or no intervention in patients with osteoarthritis of the hip or knee.

**SEARCH STRATEGY:**

We searched CENTRAL, MEDLINE (Medical Literature Analysis and Retrieval System), EMBASE (Excerpta Medica Databses), and CINAHL (Cumulative Index to Nursing and Allied Health Literature) (up to 28 July 2008), checked conference proceedings, reference lists, and contacted authors.

**SELECTION CRITERIA:**

Studies were included if they were randomised or quasi-randomised controlled trials that compared oral or transdermal opioids with placebo or no treatment in patients with osteoarthritis of the knee or hip. Studies of tramadol were excluded. No language restrictions were applied.

**DATA COLLECTION AND ANALYSIS:**

We extracted data in duplicate. Standardised mean differences (SMDs) and 95% confidence intervals (CI) were calculated for pain and function, and risk ratios for safety outcomes. Trials were combined using inverse-variance random-effects meta-analysis.

**MAIN RESULTS:**

Ten trials with 2268 participants were included. Oral codeine was studied in three trials, transdermal fentanyl and oral morphine in one trial each, oral oxycodone in four, and oral oxymorphone in two trials. Overall, opioids were more effective than control interventions in terms of pain relief (SMD -0.36, 95% CI -0.47 to -0.26) and improvement of function (SMD -0.33, 95% CI -0.45 to -0.21). We did not find substantial differences in effects according to type of opioid, analgesic potency (strong or weak), daily dose, duration of treatment or follow up, methodological quality of trials, and type of funding. Adverse events were more frequent in patients receiving opioids compared to control. The pooled risk ratio was 1.55 (95% CI 1.41 to 1.70) for any adverse event (4 trials), 4.05 (95% CI 3.06 to 5.38) for dropouts due to adverse events (10 trials), and 3.35 (95% CI 0.83 to 13.56) for serious adverse events (2 trials). Withdrawal symptoms were more severe after fentanyl treatment compared to placebo (SMD 0.60, 95% CI 0.42 to 0.79; 1 trial).

**AUTHORS’ CONCLUSIONS:**

The small to moderate beneficial effects of non-tramadol opioids are outweighed by large increases in the risk of adverse events. Non-tramadol opioids should therefore not be routinely used, even if osteoarthritic pain is severe.

FURTHER INFORMATION:

Centro Cochrane do Brasil

Rua Pedro de Toledo, 598

Vila Clementino - São Paulo (SP) - Brasil

CEP 04039-001

Tel. (+55 11) 5579-0469/5575-2970

E-mail: cochrane.dmed@epm.br

http://www.centrocohranedobrasil.org.br/

